# A Phase I open-label study to assess the pharmacokinetics, safety, and tolerability of capivasertib alone or in combination with paclitaxel in Chinese patients with advanced solid tumors

**DOI:** 10.1186/s12885-025-14982-4

**Published:** 2025-10-14

**Authors:** Jian Zhang, Xiaojun Liu, Yiqun Du, Yuxin Mu, Yanchun Meng, Yan Sun, Ling Zhang, Chris Chen, Marie Cullberg, Ethan Fan, Xichun Hu

**Affiliations:** 1 Shanghai Cancer Center, Fudan University, Shanghai, China; 2 Department of Oncology, Shanghai Medical College, Fudan University, Shanghai, China; 3 Clinical Pharmacology, Development Science, AstraZeneca R&D, Shanghai, China; 4 Oncology R&D, AstraZeneca, Shanghai China; 5 Clinical Pharmacology & Quantitative Pharmacology, Biopharmaceuticals R&D, AstraZeneca, Gothenburg, Sweden

**Keywords:** Capivasertib, Chinese, Paclitaxel, Pharmacokinetics, Phase I, Safety

## Abstract

**Background:**

Capivasertib is recommended, plus fulvestrant, for hormone receptor-positive/human epidermal growth factor receptor 2-negative advanced breast cancer with *PIK3CA*/*AKT1*/*PTEN* alterations and is under development for further breast and prostate cancer indications. Pharmacokinetics in Western and Japanese patients have been previously characterized. We conducted a Phase I trial assessing the pharmacokinetics and safety of capivasertib, alone or plus paclitaxel, in Chinese patients with advanced solid tumors.

**Methods:**

In this open-label, fixed-sequence Phase I trial, Chinese patients with advanced solid tumors refractory or resistant to standard therapy received capivasertib alone (Part A) and then plus paclitaxel (Part B). The primary endpoint comprised capivasertib pharmacokinetics after a single dose (480 mg), or multiple doses given alone (480 mg twice daily [4 days on, 3 days off]) or plus paclitaxel (capivasertib 400 mg twice daily [4 days on, 3 days off], paclitaxel 80 mg/m^2^ once weekly; both 3 weeks on, 1 week off). Safety was a secondary endpoint. Investigator-assessed best objective response was an exploratory endpoint.

**Results:**

Overall, 16 patients (median age 55.5 years, median weight 58.9 kg, 81.3% breast primary tumor location) received capivasertib alone in Part A and then capivasertib plus paclitaxel in Part B. The median time to maximum concentration, the geometric mean maximum plasma concentration, and the geometric mean area under the curve from time 0 to the last quantifiable concentration of capivasertib were: after a single dose (*n* = 16): 1.0 h, 1465 ng/mL, and 7243 h×ng/mL; after multiple doses (*n* = 15): 0.9 h, 2535 ng/mL, and 12,080 h×ng/mL; after multiple doses plus paclitaxel (*n* = 8): 1.9 h, 2467 ng/mL, and 12,830 h×ng/mL, respectively. After a single dose, the geometric mean terminal elimination half-life was 9.7 h. Hyperglycemia, diarrhea, and rash were the most common adverse events (reported for all patients). Most adverse events were Grade 1–2. Four (25.0%) patients achieved confirmed partial response and four (25.0%) stable disease as best objective response.

**Conclusions:**

Consistent with previous findings, capivasertib was absorbed rapidly and eliminated with a half-life of approximately 10 h. The manageable safety profile and preliminary antitumor activity support further investigation of capivasertib-containing combinations in Chinese patients with advanced solid tumors.

**Trial registration:**

The trial was registered at ClinicalTrials.gov with identifier no. NCT04742036. Date of registration: February 4, 2021.

**Supplementary Information:**

The online version contains supplementary material available at 10.1186/s12885-025-14982-4.

## Background

Dysregulation of the phosphatidylinositol 3-kinase (PI3K)/AKT serine/threonine kinase (AKT) pathway is a hallmark of many human malignancies, including breast and prostate cancers [1, 2]. Common mechanisms of pathway dysregulation include activating mutations in the phosphatidylinositol-4,5-bisphosphate 3-kinase catalytic subunit alpha (*PIK3CA*) or *AKT1* or loss of expression of phosphatase and tensin homolog (PTEN) [3]. The critical node of the pathway is AKT, a serine/threonine kinase that exists in three isoforms (AKT1/2/3) and phosphorylates downstream targets to promote cell proliferation and survival [4]. In patients with cancer, activation of the PI3K/AKT pathway is associated with poor prognosis and resistance to chemotherapy or endocrine therapy [5, 6].

Capivasertib is an oral, potent, and selective inhibitor of all three AKT isoforms (AKT1/2/3) [7]. In the primary analysis of the randomized Phase III CAPItello-291 trial, capivasertib plus fulvestrant significantly improved progression-free survival (PFS) compared with placebo plus fulvestrant in patients with hormone receptor (HR)-positive/human epidermal growth factor receptor 2 (HER2)-negative advanced breast cancer after recurrence or progression on an aromatase inhibitor, with or without a cyclin-dependent kinase 4/6 (CDK4/6) inhibitor [8]. PFS was significantly improved both in the overall study population (hazard ratio: 0.60, 95% confidence interval [CI]: 0.51–0.71, *p* < 0.001) and in the population of patients with *PIK3CA*/*AKT1*/*PTEN*-altered tumors (hazard ratio: 0.50, 95% CI: 0.38–0.65, *p* < 0.001) [8]. In line with the results in the global population, exploratory analysis in an extended Chinese cohort (*N* = 134) showed a clinically meaningful PFS benefit of capivasertib plus fulvestrant compared with placebo plus fulvestrant in the same setting (overall population: hazard ratio: 0.51, 95% CI: 0.34‍–‍0.76; patients with *PIK3CA*/*AKT1*/*PTEN*-altered tumors: hazard ratio: 0.41, 95% CI: 0.19–0.85) [9]. Clinical guidelines by the China Anti-Cancer Association and the United States National Comprehensive Cancer Network recommend the use of capivasertib plus fulvestrant in certain patients with HR-positive/HER2-negative advanced breast cancer with *PIK3CA/AKT1/PTEN* tumor alterations after disease progression or recurrence following one or more prior lines of endocrine therapy [10, 11].

A second Phase III trial (CAPItello-292) in patients with HR-positive/HER2-negative advanced breast cancer is evaluating capivasertib plus a CDK4/6 inhibitor (palbociclib or ribociclib) and fulvestrant after progression or recurrence on (neo)adjuvant endocrine therapy [12]. Capivasertib in combination with paclitaxel was investigated in the Phase III CAPItello-290 trial in patients with locally advanced or metastatic triple-negative breast cancer who were previously untreated in the advanced setting [13]. Capivasertib plus paclitaxel versus placebo plus paclitaxel did not meet the predefined threshold for improving the dual primary endpoints of overall survival in the overall population or in patients with *PIK3CA/AKT1/PTEN*-altered tumors, although the secondary endpoint of PFS numerically favored capivasertib plus paclitaxel [13]. Finally, Phase III trials in patients with metastatic prostate cancer are evaluating capivasertib plus abiraterone for hormone-sensitive disease with PTEN deficiency (CAPItello-281) [14] and capivasertib plus docetaxel for castration-resistant disease (CAPItello-280) [15].

Preceding the initiation of the Phase III trials, Phase I/II trials characterized the safety and pharmacokinetics (PK) of capivasertib, alone or in combination with other anticancer agents, in healthy volunteers or patients with advanced solid tumors [16–26]. In PK analyses of Western and Japanese patients, capivasertib showed rapid absorption after oral administration, with a median time to maximum plasma concentration (t_max_) of approximately 1–2 h and a terminal elimination half-life (t_½λz_) of approximately 10 h [16, 21, 22]. Plasma exposure, assessed as area under the plasma concentration–time curve (AUC) over the dosing interval and maximum plasma concentration (C_max_), was approximately dose-proportional in doses ranging from 80 mg to 800 mg [16, 22]. The recommended dose regimen for capivasertib was established as 480 mg twice daily (BD; 4 days on, 3 days off) when given as monotherapy [16], 400 mg BD (4 days on, 3 days off) in Weeks 1–3 of each 4-week cycle when given in combination with paclitaxel [17], 400 mg BD (4 days on, 3 days off) when given in combination with fulvestrant [18] or abiraterone [19], and 320 mg BD (4 days on, 3 days off) when given in combination with docetaxel (75 mg/m^2^ on Day 1 of each 21-day cycle for 10 cycles with prednisolone 5 mg BD [Days 1–21]) [20]. Capivasertib can be administered with or without food [23, 27].

A population PK (popPK) model was developed to quantitatively assess the impact of intrinsic and extrinsic factors on the PK of capivasertib. The model was based on four Phase I and II trials that used a range of capivasertib dosing schedules, either as monotherapy or in combination with fulvestrant or paclitaxel. The PK of capivasertib was only moderately altered by covariates such as body weight, dose, food effect, and combination with paclitaxel, suggesting no need for a priori dose adjustments for capivasertib [28].

Across Phase I monotherapy trials in Western and Japanese patients, capivasertib was generally well tolerated, with diarrhea, nausea, rash, and hyperglycemia among the most common adverse events [16, 21, 22]. Capivasertib also showed favorable tolerability in combination with paclitaxel, with no impact on paclitaxel tolerability or dose intensity [17, 29]. The Phase I trials exploring the PK and safety of capivasertib recruited in sites in Europe [16, 21], North America [16], and Japan [22]; however, Phase I data in Chinese patients are limited.

We conducted a Phase I trial to evaluate the PK, safety, and tolerability of capivasertib, alone or in combination with paclitaxel, in Chinese patients with advanced solid tumors.

## Methods

### Study design

**Fig. 1:**
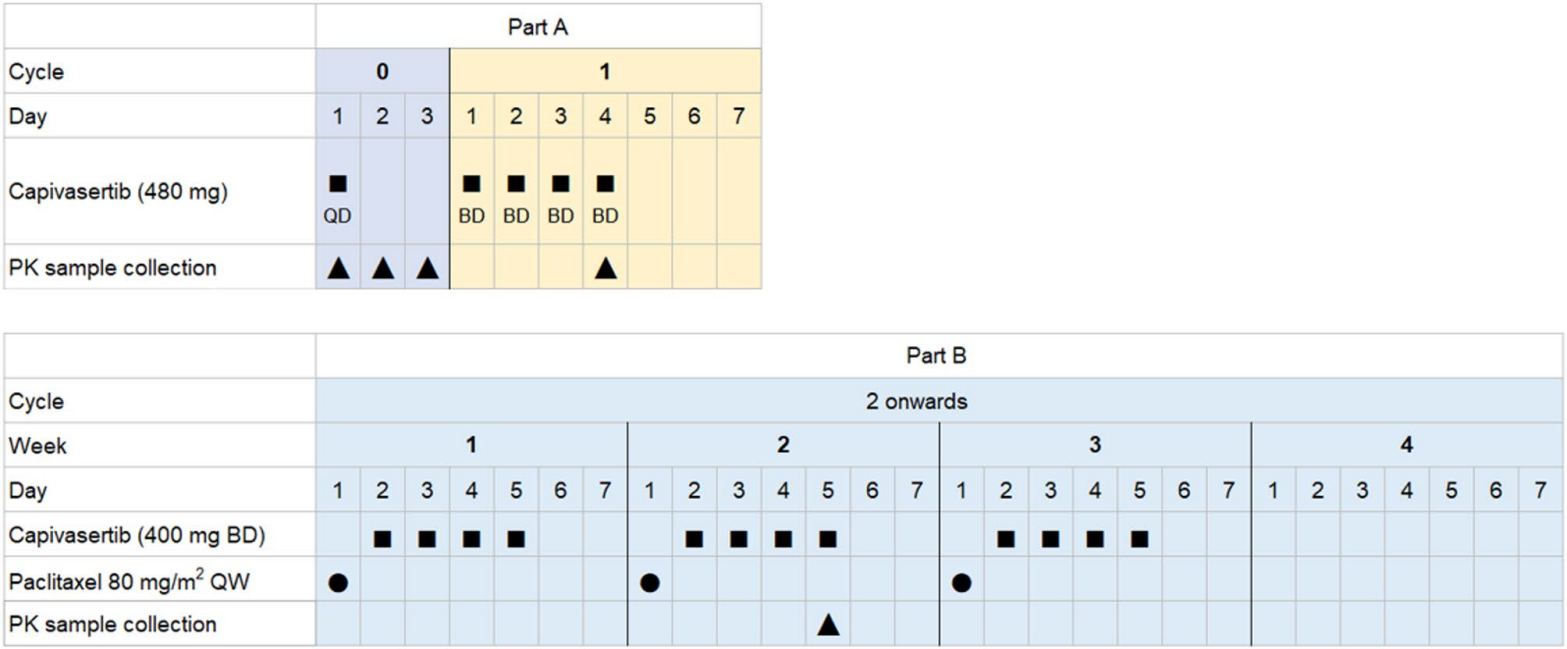
Study design. *BD* twice daily, *PK* pharmacokinetics, *QD* once daily, *QW* once weekly

This open-label, fixed-sequence, two-part Phase I study (NCT04742036) was designed to assess the PK, safety, and tolerability of capivasertib when administered alone (in Part A) and then in combination with paclitaxel (in Part B) in Chinese patients with advanced solid tumors (Fig. 1). The study was performed in accordance with the ethical principles that have their origin in the Declaration of Helsinki and that are consistent with International Council for Harmonisation/Good Clinical Practice, applicable regulatory requirements, and the sponsor policy on bioethics. All patients provided signed informed consent before admission to the study.

### Study population

Chinese patients aged ≥ 18 years took part in the study. One of the following was required for inclusion: a) ≥ 1 lesion, not previously irradiated, that could be measured accurately at baseline with computed tomography (CT) or magnetic resonance imaging (MRI) as ≥ 10 mm in the longest diameter (or ≥ 15 mm in the short axis for lymph nodes) and was suitable for accurate repeated measurements; or b) lytic or mixed (lytic plus sclerotic) bone lesions that could be assessed by CT or MRI in the absence of measurable disease as defined above; patients with sclerotic/osteoblastic bone lesions only in the absence of measurable disease were not eligible.

Patients also needed to meet the following criteria: a) histologically or, where appropriate, cytologically confirmed malignant solid tumor refractory or resistant to standard therapy and for which no suitable effective standard therapy existed; b) life expectancy of ≥ 12 weeks; and c) stable concomitant medication regimen, defined as no changes in medication or in dose within 2 weeks prior to start of capivasertib dosing, except for bisphosphonates, denosumab, and corticosteroids, which had to be stable for at least 4 weeks prior to start of capivasertib dosing.

### Endpoints

The primary endpoint comprised PK parameters for capivasertib after a single dose or multiple doses when given orally alone or after multiple doses in combination with paclitaxel. The secondary endpoints included safety and tolerability endpoints, such as reporting of adverse events and serious adverse events. Investigator-assessed best objective response per Response Evaluation Criteria in Solid Tumours (RECIST) v1.1 was an exploratory endpoint.

### Treatments

In Part A, a single dose of capivasertib 480 mg was administered orally on Cycle 0 Day 1 (Fig. 1). From Cycle 1 Day 1, patients received multiple doses of oral capivasertib on an intermittent schedule (480 mg BD, 4 days on, 3 days off for 7 days).

After completion of Part A, patients continued to Part B of the study. Within each 4-week cycle, patients received a single intravenous infusion of paclitaxel 80 mg/m^2^ once weekly (QW) on Day 1 of Weeks 1–3 (3 weeks on, 1 week off) and orally administered capivasertib 400 mg BD on Days 2–5 (4 days on, 3 days off) of Weeks 1–3 (3 weeks on, 1 week off). It was recommended that capivasertib should be taken in a fasted state (only water allowed from at least 2 h before to at least 1 h after the dose). Treatment with capivasertib continued until disease progression or any other treatment discontinuation criterion was met (Additional file 1, Supplementary methods). Treatment with paclitaxel continued for at least six cycles unless the patient experienced unacceptable paclitaxel-related toxicity or disease progression. In cases where paclitaxel treatment was terminated for reasons other than disease progression, it was recommended, at the investigator’s discretion, to administer capivasertib on Days 2–5 of every week (Weeks 1–4) of each 4-week cycle.

### Assessments

In Part A, PK samples were collected from Cycle 0 Day 1 (pre-dose and at 0.5, 1, 2, 4, 6, 8, 12, 24, and 48 h post-dose) to characterize the single-dose PK profile and on Cycle 1 Day 4 (pre-dose and at 0.5, 1, 2, 4, 6, 8, and 12 h post-dose) to characterize the multiple-dose PK profile of capivasertib in a dose interval (Fig. 1). In Part B, PK samples were collected on Cycle 2 Week 2 Day 5 (pre-dose and at 0.5, 1, 2, 4, 6, 8, and 12 h post-dose) to characterize the multiple-dose PK profile of capivasertib in a dose interval in combination with paclitaxel. For patients with dose interruption in Cycle 2 before Week 2 Day 5, sample collection could be postponed to Week 2 Day 5 of the subsequent or later cycle, providing there was no dose interruption before Week 2 Day 5 of that cycle.

Adverse events were recorded continuously until 30 (± 7) days after discontinuation of study treatment and graded using the National Cancer Institute Common Terminology Criteria for Adverse Events v5.0.

Tumor assessments per RECIST v1.1 were based on images from CT or MRI of the chest, abdomen, and pelvis (with additional anatomy as clinically indicated by the extent of disease) collected during baseline and at regular intervals during Part B of the study. Baseline assessment was performed within 28 days before the start of the study intervention. In Part B, assessments were performed every 8 weeks (± 7 days) from Cycle 3 Week 1 Day 1 until objective radiological disease progression, patient withdrawal, or 6 months after the enrollment of the last patient.

The end of study was defined as the last visit/contact of the last patient assigned to study treatment. Completion of the last scheduled visit for each patient was considered study completion for the patient.

### Statistics

Approximately 16 Chinese patients with advanced solid tumors were planned to be treated with capivasertib as monotherapy as well as in combination with paclitaxel, with the expectation of ≥ 8 evaluable patients for the PK analysis set at study completion. No formal statistical testing was performed in this study; hence, descriptive statistics were used on the PK analysis set and safety analysis set.

The PK analysis set included all patients who received ≥ 1 dose of capivasertib and had ≥ 1 post-dose reportable concentration–time data for PK analysis. The PK data were summarized by dose scheme (capivasertib single dose 480 mg and capivasertib multiple dose 480 mg BD in Part A, and capivasertib multiple dose 400 mg BD plus paclitaxel QW in Part B). The safety analysis set included all patients who received ≥ 1 dose of capivasertib and was used for safety and preliminary efficacy analyses. Safety data were summarized descriptively from all treatment cycles combined (both Parts A and B of the study).

Best objective response was summarized by total counts and percentages of patients for each response category (complete response, partial response, stable disease, progressive disease, and not evaluable). Each patient was classified only once using the best response during Part B of the study. Complete/partial responses required confirmation.

The data cutoff for analysis took place approximately 6 months after the last patient was enrolled to allow the opportunity for all patients to complete a minimum of two disease assessments by investigator based on RECIST v1.1.

## Results

### Patients

A total of 17 Chinese patients were enrolled (Additional file 1, Supplementary Fig. 1). Of those, 16 (94.1%) patients were assigned to study treatment; one patient failed at screening due to study inclusion/exclusion criteria. All 16 patients received capivasertib monotherapy in Part A and then moved on to Part B of the study and received capivasertib in combination with paclitaxel. At baseline, the median age of the patients was 55.5 years and the median weight 58.9 kg (Table 1). Most (*n* = 14 [87.5%]) patients were female, and all patients had Eastern Cooperative Oncology Group performance status (ECOG PS) score 1. The primary tumor location was breast for most (*n* = 13 [81.3%]) patients and all patients had metastatic disease at study baseline.

**Table 1:** Baseline characteristics

Characteristic	Safety analysis set (*N* = 16)
Age (years), median (range)	55.5 (42–72)
Age group (years), *n* (%)	
< 50	5 (31.3)
≥ 50–<65	9 (56.3)
≥ 65–<75	2 (12.5)
Sex, *n* (%)	
Female	14 (87.5)
Male	2 (12.5)
Race, *n* (%)	
Asian	16 (100.0)
Weight (kg), median (range)	58.9 (46.8–74.6)
ECOG PS, *n* (%)	
1	16 (100.0)
Primary tumor location, *n* (%)	
Breast	13 (81.3)
Nasopharynx	1 (6.3)
Prostate gland	1 (6.3)
Urethra	1 (6.3)
Tumor histology types, *n* (%)	
Invasive carcinoma	3 (18.8)
Invasive ductal carcinoma	3 (18.8)
Invasive ductal carcinoma with extensive intraductal component	3 (18.8)
Acinar adenocarcinoma	1 (6.3)
Urothelial carcinoma	1 (6.3)
Other	5 (31.3)
Previous treatments, *n* (%)	
Cytotoxic chemotherapy	16 (100)
Hormonal therapy	11 (68.8)
Targeted therapy	10 (62.5)
Radiotherapy	7 (43.8)
Immunotherapy	3 (18.8)
Other	2 (12.5)

*ECOG PS* Eastern Cooperative Oncology Group performance status

Among the 16 patients, 12 (75.0%) completed the study, whereas four (25.0%) did not complete the study (one [6.3%] patient died before the safety follow-up; three [18.8%] patients did not complete the final visit of the study by the data cutoff date. Of those, two [12.5%] patients had not progressed and one [6.3%] patient had not completed the safety follow-up by the data cutoff date). At the time of data cutoff (July 29, 2022), two (12.5%) patients were still under treatment: one (6.3%) patient receiving capivasertib and paclitaxel and one (6.3%) patient receiving capivasertib only.

### Pharmacokinetics

In Part A, PK samples were collected from all 16 patients for single-dose assessment and from 15 patients for multiple-dose assessment of capivasertib alone. Samples for the multiple-dose assessment were not collected from one patient due to dose interruption prior to the collection date.

In Part B, PK samples were collected from 10 patients for multiple-dose assessment of capivasertib in combination with paclitaxel. Disease progression in six patients precluded the collection of samples before a suitable predefined collection date (i.e. Week 2 Day 5 of Cycle 2 or later cycle, providing there was no dose interruption before Week 2 Day 5 of that cycle). Descriptive summary statistics are presented for eight patients, as two additional patients were excluded due to capivasertib dose reduction from 400 mg BD to 320 mg BD prior to the sample collection period in Cycle 2.

**Fig. 2:**
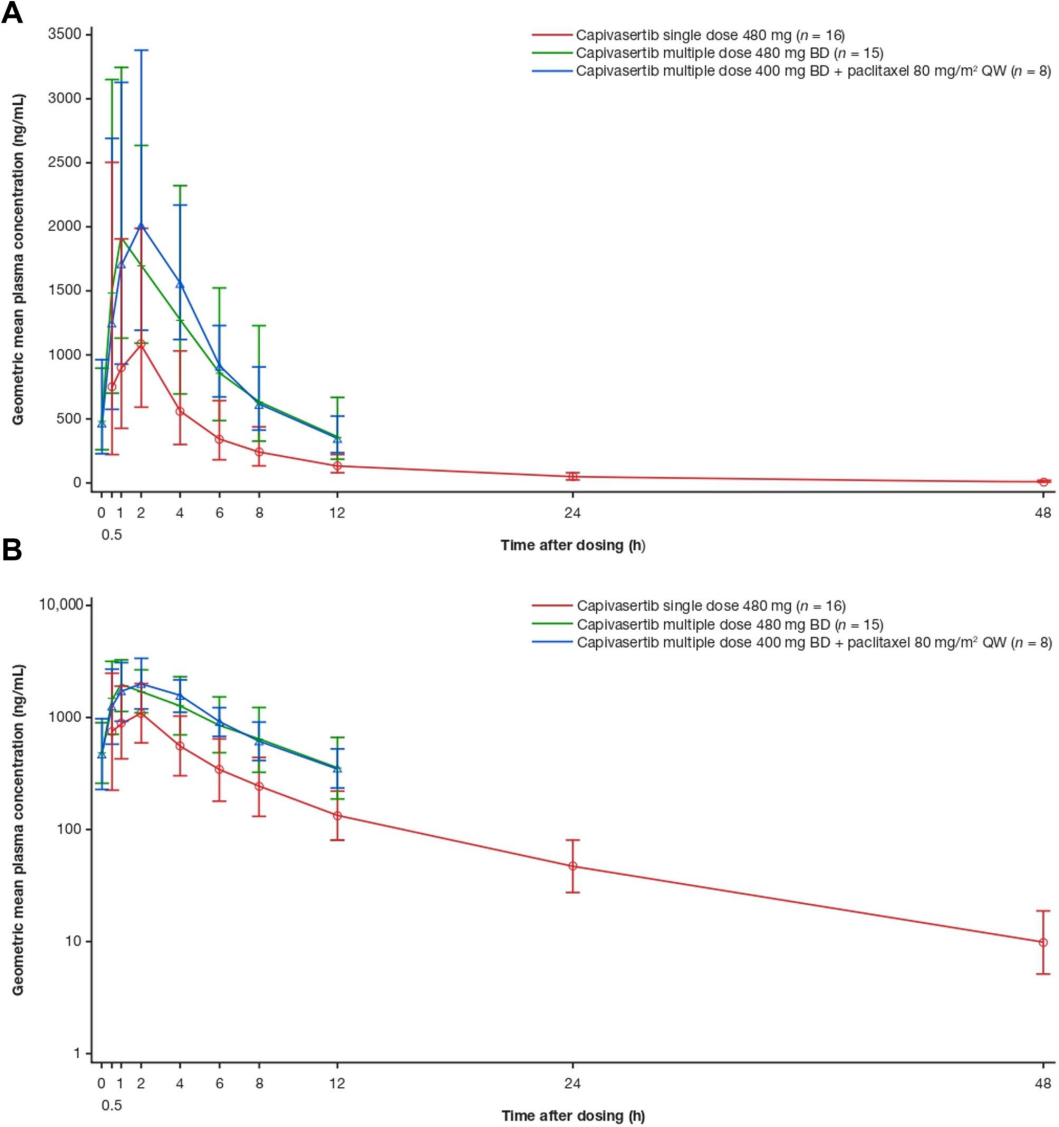
Capivasertib plasma concentrations over time on an (A) linear or (B) semi-logarithmic scale. Points and error bars show the geometric mean ± geometric standard deviation of plasma concentrations of capivasertib (single dose 480 mg, multiple dose 480 mg BD, or multiple dose 400 mg BD in combination with paclitaxel) over time. Patients received capivasertib single dose 480 mg on Cycle 0 Day 1, capivasertib multiple dose 480 mg on Cycle 1 Day 4, and capivasertib multiple dose 400 mg BD on Cycle X Week 2 Day 5, where X can be Cycle 2 or laterBD twice daily, QW once week

A similar shape of capivasertib plasma concentration–time profile was observed after single and multiple doses of capivasertib alone and multiple doses of capivasertib in combination with paclitaxel (Fig. 2). Capivasertib was eliminated in an approximately biphasic manner in all instances.

After a single dose of capivasertib 480 mg, all patients (*n* = 16) had measurable capivasertib plasma concentrations up to 48 h post-dose, the median t_max_ was 1.0 h (range: 0.4–4.0), and the geometric mean t_½λz_ was 9.7 h (geometric coefficient of variation [gCV]: 12.7%). Additionally, the systemic exposure in terms of geometric mean C_max_ and AUC from time 0 to the last quantifiable concentration (AUC_last_) was 1465 ng/mL (gCV: 72.5%) and 7243 h×ng/mL (gCV: 58.8%), respectively (Table 2), while the geometric mean apparent total body clearance (CL/F) was 64.9 L/h (gCV: 58.7%; Table 2).

**Table 2:** Capivasertib pharmacokinetic parameters when administered alone or plus paclitaxel

Parameter	Statistic	Capivasertib single dose 480 mg (*n* = 16)	Capivasertib multiple dose 480 mg (*n* = 15)	Capivasertib 400 mg plus paclitaxel multiple dose (*n* = 8)
C_max_ (ng/mL)	gMean gCV, %	1465 72.5	2535 48.7	2467 48.5
AUC_inf_ (h×ng/mL)	gMean gCV, %	7397 58.7	–	–
AUC_(0–12)_ (h×ng/mL)	gMean gCV, %	5630 62.2	12,120 49.3	12,860 32.0
AUC_(0–48)_ (h×ng/mL)	gMean gCV, %	7244 58.8	–	–
AUC_last_ (h×ng/mL)	gMean gCV, %	7243 58.8	12,080 49.2	12,830 32.0
t_max_ (h)	Median (range)	1.0 0.4–4.0	0.9 0.4–3.9	1.9 0.5–4.0
t_½λz_ (h)	gMean gCV,%	9.7 12.7	–	–
t_last_ (h)	Median (range)	47.9 47.9–48.1	11.9 11.8–12.0	11.9 11.9–12.0
CL/F (L/h)	gMean gCV, %	64.9 58.7	39.6 49.3	31.1 32.0
RacC_max_	gMean gCV, %	–	1.8 60.5	–
RacAUC_(0–12)_	gMean gCV, %	–	2.3 50.3	–
TCP	gMean gCV, %	–	1.8 42.8	–

Capivasertib single dose 480 mg: patients received a single dose of capivasertib 480 mg only on Cycle 0 Day 1. Capivasertib multiple dose 480 mg: patients received capivasertib 480 mg twice daily (4 days on, 3 days off). Capivasertib 400 mg multiple dose plus paclitaxel: patients received capivasertib 400 mg (4 days on, 3 days off) and paclitaxel 80 mg/m^2^ on Weeks 1–3 of each 4-week cycle, respectively

*AUC*_*(0–12)*_ area under the plasma concentration–time curve from time 0 to 12 h post-dose, *AUC*_*(0–48)*_ area under the plasma concentration–time curve from time 0 to 48 h post-dose, *AUC*_*inf*_ area under the plasma concentration–time curve from time 0 to infinity, *AUC*_*last*_ area under the plasma concentration–time curve from time 0 to the last quantifiable concentration, *CL/F* apparent total body clearance, *C*_*max*_ maximum plasma concentration, *gCV* geometric coefficient of variation, *gMean* geometric mean, *RacAUC*_*(0–12)*_ accumulation ratio for AUC_(0–12)_, *RacC*_*max*_ accumulation ratio for C_max_, *t*_*½λz*_ terminal elimination half-life, *TCP* temporal change parameter (AUC_[0–12]_, capivasertib multiple dose 480 mg/AUC_inf_, capivasertib single dose 480 mg), *t*_*last*_ time of last quantifiable plasma concentration, *t*_*max*_ time to maximum plasma concentration

After multiple doses of capivasertib 480 mg BD, all evaluable patients (*n* = 15) had measurable capivasertib plasma concentrations up to 12 h post-dose, the median t_max_ was 0.9 h (range: 0.4–3.9), and the systemic exposure in terms of geometric mean C_max_ and AUC_last_ was 2535 ng/mL (gCV: 48.7%) and 12,080 h×ng/mL (gCV: 49.2%), respectively (Table 2). Additionally, the geometric mean CL/F was 39.6 L/h (gCV: 49.3%; Table 2). Finally, the geometric mean accumulation ratios for C_max_ (RacC_max_) and AUC from time 0 to 12 h post-dose (RacAUC_[0–12]_) were 1.8 (gCV: 60.5%) and 2.3 (gCV: 50.3%), respectively, indicating approximately two-fold accumulation of capivasertib over time (Table 2), while the geometric mean temporal change parameter (AUC_[0–12]_, capivasertib multiple dose 480 mg/AUC from time 0 to infinity, capivasertib single dose 480 mg; TCP) value was 1.8 (gCV: 42.8%), suggesting time-dependent PK (Table 2).

After multiple doses of capivasertib 400 mg BD (4 days on, 3 days off) in combination with paclitaxel 80 mg/m^2^ QW (3 weeks on, 1 week off for both treatments), the eight patients included in summary statistics all had measurable capivasertib plasma concentrations up to 12 h post-dose (Fig. 2). The capivasertib median t_max_ was 1.9 h (range: 0.5–4.0), and systemic exposure in terms of geometric mean C_max_ and AUC_last_ was 2467 ng/mL (gCV: 48.5%) and 12,830 h×ng/mL (gCV: 32.0%), respectively (Table 2). Additionally, the geometric mean CL/F was 31.1 L/h (gCV: 32.0%; Table 2).

### Safety

In Part A, the median (range) total treatment duration for capivasertib was 1 day (1–1) for Cycle 0, as all patients were expected to receive one single dose, and 4 days (3–4) for Cycle 1. In Part B, the median (range) total treatment duration was 20.6 weeks (2.6–48.1) for capivasertib and 15.0 weeks (3.0–46.6) for paclitaxel.

The safety analysis set included all patients who received ≥ 1 dose of capivasertib (*N* = 16), and the results presented derive from all the treatment cycles combined (both Part A and B). Overall, hyperglycemia, diarrhea, and rash were the most common adverse events and were reported for all patients (Table 3); they were also the most common adverse events considered as possibly related to capivasertib only (Additional file 1, Supplementary Table 1). Most adverse events reported were low grade (Grade 1–2). The most common adverse events of Grade ≥ 3 were neutrophil count decreased, experienced by 12 (75.0%) patients, and white blood cell count decreased, experienced by 10 (62.5%) patients (Table 3). Three serious adverse events were reported for two (12.5%) patients: one patient experienced device-related infection (catheter) and hyperglycemia, and the other patient febrile neutropenia. All three events were considered to be possibly related to both capivasertib and paclitaxel. No patients died due to adverse events or experienced adverse events leading to discontinuation of any study treatment. Adverse events led to dose interruptions of capivasertib in 14 (87.5%) patients and dose reductions of capivasertib in five (31.3%) patients. Adverse events led to dose interruptions of paclitaxel in 12 (75.0%) patients and dose reductions of paclitaxel in five (31.3%) patients.

**Table 3:** Most frequent adverse events in patients who received capivasertib and paclitaxel

Adverse event	Patients (*N* = 16), *n* (%)	
	Any grade	Grade ≥ 3
Any adverse event	16 (100.0)	13 (81.3)
Diarrhoea	16 (100.0)	0
Hyperglycaemia	16 (100.0)	3 (18.8)
Rash^a^	16 (100.0)	5 (31.3)
Neutrophil count decreased	15 (93.8)	12 (75.0)
White blood cell count decreased	15 (93.8)	10 (62.5)
Anaemia	13 (81.3)	2 (12.5)
Hyponatraemia	10 (62.5)	0
Hypophosphataemia	10 (62.5)	0
Pyrexia	9 (56.3)	1 (6.3)
Stomatitis	8 (50.0)	0
Vomiting	8 (50.0)	0
Blood creatinine increased	7 (43.8)	0
Hypokalaemia	7 (43.8)	1 (6.3)
Proteinuria	7 (43.8)	0
Weight decreased	7 (43.8)	0
Aspartate aminotransferase increased	6 (37.5)	0
Blood albumin decreased	6 (37.5)	0
Hypomagnesaemia	6 (37.5)	0
Oedema peripheral	6 (37.5)	0
Peripheral sensory neuropathy	6 (37.5)	0
Glycosylated haemoglobin increased	5 (31.3)	0
Hypercalcaemia	5 (31.3)	0
Hypoalbuminaemia	5 (31.3)	0
Hypocalcaemia	5 (31.3)	1 (6.3)
Fatigue	5 (31.3)	1 (6.3)
Influenza-like illness	5 (31.3)	0
Nausea	5 (31.3)	0
Blood bilirubin increased	4 (25.0)	0
Blood triglycerides increased	4 (25.0)	0
Blood uric acid increased	4 (25.0)	0
Malaise	4 (25.0)	0
Platelet count decreased	4 (25.0)	0
Pruritus	4 (25.0)	0

Parts A and B of the study combined. Adverse events occurring in ≥ 25% of the safety analysis set. Adverse events with an onset date on/after the date of the first dose of study treatment and adverse events with an onset date prior to the first dose that worsen after the first dose are reported up to 30 days (+ 7 days) following the date of last dose or data cutoff (whichever is earlier). The safety analysis set included all patients who received at least one dose of capivasertib

^a^The group term of rash comprises the preferred terms of rash, rash macular, rash maculopapular, rash papular, and rash pruritic. In this study, all the adverse events of rash reported were rash maculopapular

### Best objective response per investigator assessment

Preliminary efficacy was assessed in Part B using the safety analysis set, which included all patients who received ≥ 1 dose of capivasertib (*N* = 16). All 16 patients had measurable disease at study baseline and were classified based on the best response achieved during treatment with capivasertib in combination with paclitaxel. Four (25.0%) patients achieved confirmed partial response, whereas best objective response of stable disease was noted in four (25.0%) patients. Of those with stable disease, two had an unconfirmed partial response, i.e. a partial response with no confirmation assessment or with an assessment that did not confirm the response. Finally, seven (43.8%) patients had a best objective response of progressive disease, whereas the response status of one patient (6.3%) was not evaluable (stable disease < 7 weeks).

## Discussion

This Phase I trial was conducted to evaluate the PK and safety of capivasertib, administered with or without paclitaxel, in Chinese patients with advanced solid tumors refractory/resistant to standard therapy.

The plasma concentration–time profile of capivasertib administered as monotherapy exhibited a similar shape following a single 480 mg dose or multiple 480 mg BD doses. Capivasertib was absorbed into the circulation rapidly, with median t_max_ of approximately 1 h, and then eliminated in an approximately biphasic manner, with an estimated t_½λz_ of approximately 10 h. Compared with single doses, multiple dosing was associated with an approximately two-fold accumulation of capivasertib. Lower clearance and a time-dependent increase in exposure, based on the TCP value (1.8), were observed after multiple doses of capivasertib. These findings are consistent with the capivasertib popPK model derived from Western and Japanese patients and reported by Fernandez-Teruel et al. [28], which also showed modestly time-dependent clearance during the first week of treatment, potentially due to cytochrome P450 3A4 autoinhibition. Overall, the PK of capivasertib monotherapy after single or multiple intermittent BD doses was in line with prior observations from Phase I trials in American, European, or Japanese patients [16, 21, 22].

The capivasertib popPK model reported by Fernandez-Teruel et al. [28] was further developed to include data from the Phase I trial reported in this manuscript, as well as the Phase III CAPItello-291 global and Chinese extension cohorts (six clinical trials in total) [30]. The addition of these cohorts showed minimal impact on the parameter estimates compared with the reference model. PK of capivasertib was adequately described by a three-compartment model with moderate inter-patient variability and no major covariate effects. In line with the popPK model reported by Fernandez-Teruel et al. [28], although body weight, age, fed status, and paclitaxel use showed statistically significant (*p* < 0.001) effects on capivasertib PK parameters (CL/F, AUC, and C_max_), the effects were not expected to be clinically relevant. There was no significant effect of race or region (including China, Asia [including China], and rest of the world) on capivasertib PK. Exposure–response analyses of capivasertib in combination with fulvestrant in the CAPItello-291 trial showed no significant relationship between systemic capivasertib exposure and efficacy endpoints and no differences between the Chinese and non-Chinese cohorts. The safety analysis, which pooled data from CAPItello-291 (global and Chinese cohorts) as well as a Phase I trial in patients with advanced solid tumors, identified significant relationships between capivasertib exposure and the likelihood of the following safety endpoints studied: adverse event leading to dose modification, adverse event Grade ≥ 3, and diarrhea adverse event Grade ≥ 2 [30].

The safety and tolerability profile of capivasertib monotherapy followed by capivasertib plus paclitaxel in this population of patients with advanced solid tumors was consistent with the known safety profiles of both study drugs [17, 29]. Hematologic adverse events, stomatitis, vomiting, diarrhea, and neuropathy are known adverse events of paclitaxel [31] and were also observed in this study. Consistent with previous studies of capivasertib as monotherapy or in combination with other anticancer agents [8, 16, 17, 21, 22, 29, 32], the most common adverse events included diarrhea, hyperglycemia, and rash, which were mostly Grade 1/2 in severity. All patients in this study experienced diarrhea (0% Grade ≥ 3), hyperglycemia (18.8% Grade ≥ 3), and rash (31.3% Grade ≥ 3). In the BEECH [17] and PAKT [29] studies examining capivasertib plus paclitaxel, diarrhea was reported in 75.9% (24.1% Grade ≥ 3) and 72.1% (13.2% Grade ≥ 3) of patients, hyperglycemia in 29.6% (13.0% Grade ≥ 3) and 8.8% (1.5% Grade ≥ 3) of patients, and rash in 25.9% (maculopapular rash; 9.3% Grade ≥ 3) and 41.2% (4.4% Grade ≥ 3) of patients, respectively.

The higher incidence of these common adverse events in our cohort of Chinese patients relative to prior studies of capivasertib plus paclitaxel in Western patients with advanced breast cancer [17, 29] may be explained by the enrollment of a more heavily pretreated population with advanced solid tumors and poorer ECOG PS. However, comparisons should be made with caution, as the PAKT [29] and BEECH [17] studies had different study designs and recruited a larger population compared with the current study. Ethnic differences cannot be excluded; prior studies examining alpelisib have suggested that PI3K/AKT inhibitors may have ethnicity-related differences in toxicity incidence, in particular cutaneous reactions and hyperglycemia [33–35]. In the randomized CAPItello-291 trial of capivasertib plus fulvestrant versus placebo plus fulvestrant, higher incidence of low-grade hyperglycemia and other laboratory-based adverse events were observed in the extended Chinese cohort compared with the global CAPItello-291 population [8, 9]. That said, higher incidence was reported in both trial arms (capivasertib plus fulvestrant and placebo plus fulvestrant) and could also be attributable to differences in baseline characteristics or other unknown factors [9]. Based on the present data, clear conclusions regarding the source of the discrepancies in reported adverse events in these cohorts of Chinese patients versus other populations cannot be drawn. In the present study, although adverse events led to dose interruptions in capivasertib and paclitaxel in 87.5% and 75.0% of patients, respectively, and to dose reductions in capivasertib and paclitaxel in 31.3% and 31.3% of patients, respectively, no patients discontinued treatment due to adverse events, underscoring the manageable safety profile of the treatments. Overall, there were no new clinically significant findings that may alter the current understanding of the capivasertib safety profile as monotherapy or in combination with paclitaxel.

This study provides preliminary evidence of the antitumor activity of capivasertib plus paclitaxel in Chinese patients with treatment-resistant advanced solid tumors, as a partial response rate of 25.0% was noted in Part B of the study. Capivasertib plus paclitaxel was previously shown to significantly increase PFS and numerically favor overall survival compared with placebo plus paclitaxel in first-line treatment of patients with triple-negative advanced breast cancer in the randomized Phase II PAKT trial conducted in Europe and South Korea [29, 36]. In both the overall population and patients with *PIK3CA/AKT1/PTEN*-altered tumors, the global Phase III CAPItello-290 trial did not meet the predefined threshold for improving the dual primary endpoints of overall survival in patients with locally advanced or metastatic triple-negative breast cancer following first-line capivasertib plus paclitaxel versus placebo plus paclitaxel [13]. However, the secondary endpoints of PFS and objective response rate numerically favored capivasertib plus paclitaxel versus placebo plus paclitaxel in both populations [13]. Of note, although the CAPItello-290 trial was based on a global cohort of patients, including Chinese patients, an extended Chinese cohort trial is ongoing. In patients with HR-positive/HER2-negative advanced breast cancer with progression on aromatase inhibitor treatment, the Phase III CAPItello-291 trial has demonstrated improvements in PFS with capivasertib plus fulvestrant versus placebo plus fulvestrant in the overall population and in patients with *PIK3CA/AKT1/PTEN*-altered tumors in a global population and a Chinese extended cohort [8, 9]. Further Phase III data for capivasertib-containing combinations are anticipated from ongoing global trials in HR-positive/HER2-negative advanced breast cancer (capivasertib plus a CDK4/6 inhibitor and fulvestrant; CAPItello-292) [37], hormone-sensitive metastatic prostate cancer with PTEN loss (capivasertib plus abiraterone; CAPItello-281) [14], and castration-resistant metastatic prostate cancer (capivasertib plus docetaxel; CAPItello-280) [15].

Limitations of the study include the limited sample size and single-center design, as well as the analysis of samples from only eight patients in Part B of the study due to failure to collect samples before the predefined date or due to capivasertib dose reduction prior to sample collection.

## Conclusions

In conclusion, this Phase I trial provided PK and safety characterization of capivasertib as monotherapy or in combination with paclitaxel in Chinese patients with advanced solid tumors. Consistent with previous findings, capivasertib was absorbed into the circulation rapidly and eliminated with an estimated t_½λz_ of approximately 10 h. The manageable safety profile and preliminary antitumor activity observed provide further support for the investigation of capivasertib-containing combinations in Chinese patients with advanced solid tumors.

## Supplementary Information

Supplementary Material 1

## Data Availability

Data underlying the findings described in this manuscript may be obtained in accordance with AstraZeneca’s data sharing policy described at [https://www.astrazenecaclinicaltrials.com/our-transparency-commitments/]. Anonymized datasets (to General Data Protection Regulation standards, with link to patient code destroyed) would be available on request. Data could be requested through Vivli at (https://vivli.org/members/enquiries-about-studies-not-listed-on-the-vivli-platform). AstraZeneca’s Vivli member page is also available, outlining further details: (https://vivli.org/ourmember/astrazeneca). Some patients/countries may need to be excluded based on the informed consent form or country-level legislation (e.g. Chinese patients would be excluded based on Human Genetic Resources Regulations). Patients who have withdrawn consent for data use will also be removed from the shared dataset. Any data submitted to this journal will have elements removed to reduce risk of patient reidentification while meeting the journal’s needs for data reproducibility and transparency. Only clinical trial data may be shared.

## Abbreviations

AKT: AKT serine/threonine kinase
AUC: Area under the plasma concentration–time curve
AUC_(0–12)_: Area under the plasma concentration–time curve from time 0 to 12 h post-dose
AUC_(0–48)_: Area under the plasma concentration–time curve from time 0 to 48 h post-dose
AUC_inf_: Area under the plasma concentration–time curve from time 0 to infinity
AUC_last_: Area under the plasma concentration–time curve from time 0 to the last quantifiable concentration
BD: Twice daily
CDK4/6: Cyclin-dependent kinase 4/6
CI: Confidence interval
CL/F: Apparent total body clearance
C_max_: Maximum plasma concentration
CT: Computed tomography
ECOG PS: Eastern Cooperative Oncology Group performance status
gCV: Geometric coefficient of variation
gMean: Geometric mean
HER2: Human epidermal growth factor receptor 2
HR: Hormone receptor
MRI: Magnetic resonance imaging
PFS: Progression-free survival
PI3K: Phosphatidylinositol 3-kinase
*PIK3CA*: Phosphatidylinositol-4,5-bisphosphate 3-kinase catalytic subunit alpha
PK: Pharmacokinetics
PopPK: Population pharmacokinetics
PTEN: Phosphatase and tensin homolog
QD: Once daily
QW: Once weekly
RacAUC_(0–12)_: Accumulation ratio for AUC_(0–12)_
RacC_max_: Accumulation ratio for C_max_
RECIST: Response Evaluation Criteria in Solid Tumours
t_½λz_: Terminal elimination half-life
TCP: Temporal change parameter (AUC_[0–12]_, capivasertib multiple dose 480 mg/AUC_inf_, capivasertib single dose 480 mg)
t_last_: Time of last quantifiable plasma concentration
t_max_: Time to maximum plasma concentration
