# Supplementary material for: A Phase I open-label study to assess the pharmacokinetics, safety, and tolerability of capivasertib alone or in combination with paclitaxel in Chinese patients with advanced solid tumors
Source: BMC Cancer. 2025 Oct 14;25:1562. doi: 10.1186/s12885-025-14982-4 (PMC12519771; doi:10.1186/s12885-025-14982-4)

# Supplementary material

## Supplementary methods

### Criteria for discontinuation of study treatment with capivasertib

- Objective disease progression per Response Evaluation Criteria in Solid Tumours (RECIST) v1.1
- Clinical disease progression/worsening of disease under investigation
- Patients incorrectly initiated on study treatment
- Patient became pregnant
- Intercurrent illness that, in the judgment of the investigator, could affect assessments of clinical status to a significant degree or contraindicate further dosing
- Unacceptable toxicity
- Determination by the investigator that it was no longer safe for the patient or in the patient’s best interest to continue therapy
- Patient request
- Severe non-compliance with the clinical study protocol in the judgment of the investigator and/or the sponsor
- Clinical need for concomitant or ancillary therapy (i.e. non-protocol-specified anticancer therapy) that was not permitted in the study
- General or specific changes in the patient’s condition that rendered the patient’s condition unacceptable for further treatment in the judgment of the investigator

## Supplementary Table 1 Most frequent adverse events possibly related to study treatment per investigator assessment

|  | **Patients with an adverse event related to both capivasertib and paclitaxel**  **(*N* = 16), *n* (%)** | | **Patients with an adverse event related to capivasertib only**  **(*N* = 16), *n* (%)** | |
| --- | --- | --- | --- | --- |
| **Adverse event** | **Any grade** | **Grade ≥3** | **Any grade** | **Grade ≥3** |
| Any adverse event | 16 (100.0) | 12 (75.0) | 16 (100.0) | 9 (56.3) |
| Diarrhoea | 16 (100.0) | 0 | 11 (68.8) | 0 |
| Hyperglycaemia | 15 (93.8) | 3 (18.8) | 15 (93.8) | 2 (12.5) |
| Neutrophil count decreased | 15 (93.8) | 12 (75.0) | 1 (6.3) | 0 |
| White blood cell count decreased | 15 (93.8) | 10 (62.5) | 2 (12.5) | 1 (6.3) |
| Anaemia | 13 (81.3) | 2 (12.5) | 4 (25.0) | 0 |
| Hypophosphataemia | 10 (62.5) | 0 | 0 | 0 |
| Rash^a^ | 9 (56.3) | 0 | 10 (62.5) | 5 (31.3) |
| Stomatitis | 8 (50.0) | 0 | 1 (6.3) | 0 |
| Hyponatraemia | 7 (43.8) | 0 | 5 (31.3) | 0 |
| Pyrexia | 7 (43.8) | 1 (6.3) | 6 (37.5) | 0 |
| Vomiting | 7 (43.8) | 0 | 1 (6.3) | 0 |
| Weight decreased | 7 (43.8) | 0 | 0 | 0 |
| Aspartate aminotransferase increased | 6 (37.5) | 0 | 1 (6.3) | 0 |
| Blood albumin decreased | 6 (37.5) | 0 | 1 (6.3) | 0 |
| Hypokalaemia | 6 (37.5) | 1 (6.3) | 1 (6.3) | 0 |
| Oedema peripheral | 6 (37.5) | 0 | 1 (6.3) | 0 |
| Peripheral sensory neuropathy | 6 (37.5) | 0 | 0 | 0 |
| Blood creatinine increased | 5 (31.3) | 0 | 2 (12.5) | 0 |
| Glycosylated haemoglobin increased | 5 (31.3) | 0 | 0 | 0 |
| Hypocalcaemia | 5 (31.3) | 1 (6.3) | 1 (6.3) | 0 |
| Nausea | 5 (31.3) | 0 | 2 (12.5) | 0 |
| Blood bilirubin increased | 4 (25.0) | 0 | 2 (12.5) | 0 |
| Blood triglycerides increased | 4 (25.0) | 0 | 0 | 0 |
| Blood uric acid increased | 4 (25.0) | 0 | 1 (6.3) | 0 |
| Hypomagnesaemia | 4 (25.0) | 0 | 4 (25.0) | 0 |
| Platelet count decreased | 4 (25.0) | 0 | 1 (6.3) | 0 |
| Proteinuria | 4 (25.0) | 0 | 5 (31.3) | 0 |
| Hypoalbuminaemia | 3 (18.8) | 0 | 4 (25.0) | 0 |

Parts A and B of the study combined. Adverse events occurring in ≥25% of the safety analysis set for at least one of the treatments. Adverse events with an onset date on/after the date of the first dose of study treatment and adverse events with an onset date prior to the first dose that worsen after the first dose are reported up to 30 days (+7 days) following the date of last dose or data cutoff (whichever is earlier). The safety analysis set included all patients who received at least one dose of capivasertib

^a^The group term of rash comprises the preferred terms of rash, rash macular, rash maculopapular, rash papular, and rash pruritic. In this study, all the adverse events of rash reported were rash maculopapular

## Supplementary Fig. 1 Participant flow diagram


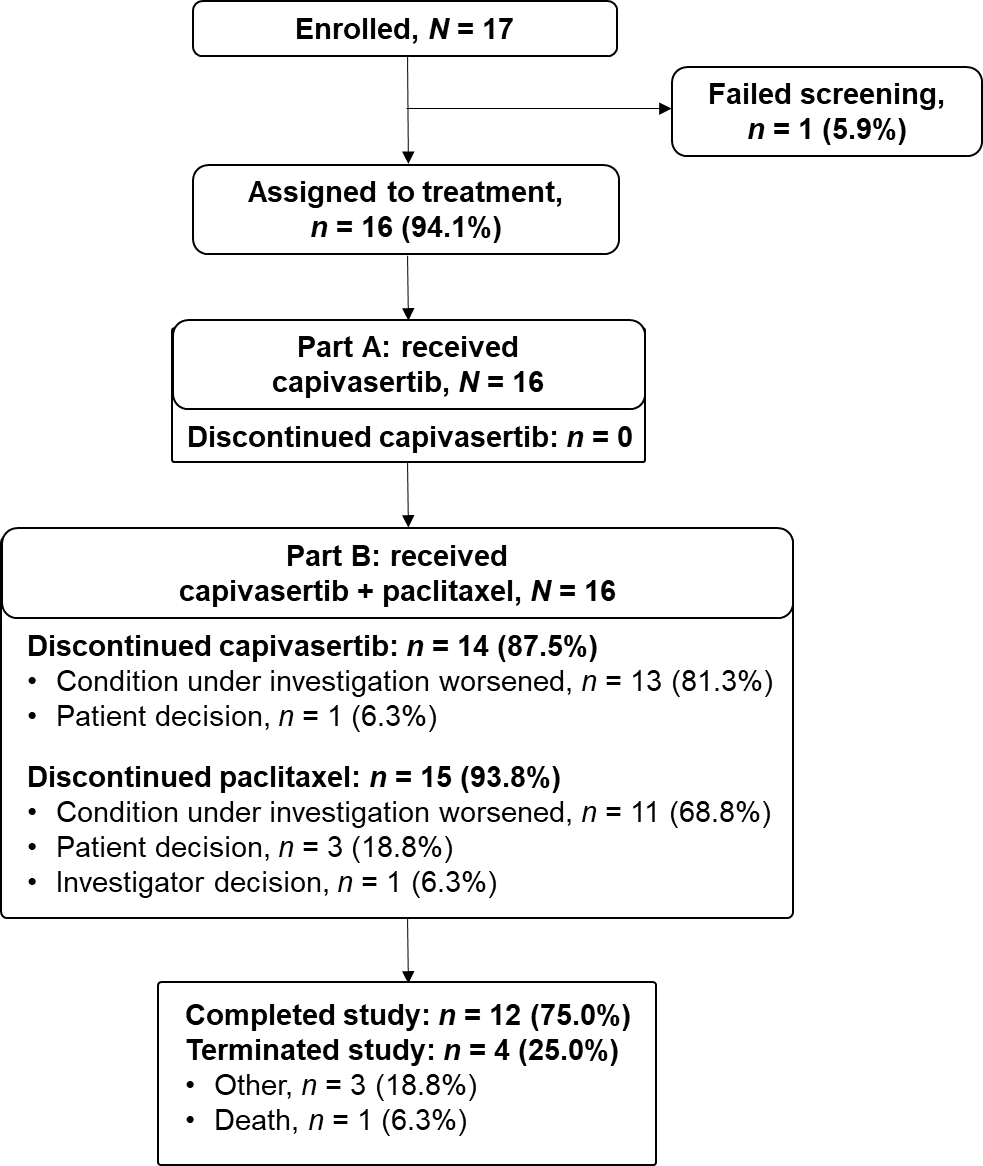

Supplement: Supplementary file 1 — Supplementary Material 1 [file 12885_2025_14982_MOESM1_ESM.docx]
